# Supplementary material for: Metabolic profiling of alcohol consumption in 9778 young adults
Source: Int J Epidemiol. 2016 Aug 5;45(5):1493–506. doi: 10.1093/ije/dyw175 (PMC5100616; doi:10.1093/ije/dyw175)
Supplement: Supplementary Data [file dyw175_supplementary_data.zip › ije-2016-02-0250-File002_coloring_corrected.pdf]

SUPPLEMENTARY APPENDIX

Würtz et al. Metabolic profiling of alcohol consumption in 9778 young adults.

Study populations.

Table S1. Mean (SD) concentrations of metabolic measures in the study population, and association magnitudes in absolute concentration units.

Figure S1. Distributions of alcohol intake in the three population-based cohorts.

Figure S2. Lipid and metabolite concentrations as a function of alcohol intake.

Figure S3. Cross-sectional associations between alcohol consumption and metabolic measures separately in the three population-based cohorts.

Figure S4. Cross-sectional associations between alcohol consumption and metabolic measures for men and women.

Figure S5: Cross-sectional associations with adjustment for potential confounders.

Study populations

The Northern Finland Birth Cohort of 1966 (NFBC-1966) was initiated to study factors affecting preterm birth and subsequent morbidity in the two northernmost provinces in Finland (www.oulu.fi/nfbc). The NFBC-1966 included n=12 058 children born into the cohort, comprising 96% of all births during 1966 in the target region. [1] Data collection in 1997 included clinical examination and serum sampling at age 31 for n=6007 individuals. Attendees in the 31-year field study (52%) were representative of the original cohort. [1] Metabolic profiling and alcohol data from this time point were analysed in the present study. In total, n=5711 persons provided blood samples (96% were at least 8h fasting), and the resulting serum samples were stored at -80°C prior to metabolic profiling by NMR metabolomics.[2] After exclusion of individuals missing data on alcohol consumption (n=686) or alcohol intake in the highest percentile (n=37) as well as pregnant women (n=151), n=4837 participants were included for the present analyses. Physical activity index was calculated as metabolic-equivalent-of-task, based on questionnaire data on frequency, intensity, and duration of physical activity.[3] Current smoking was assessed from questionnaires. In addition to NMR-based metabolic profiling, plasma levels of the following protein biomarkers were measured by standard clinical chemistry assays and analysed in the present study: C-reactive protein, alanine aminotransferase, gamma-glutamyl aminotransferase, and plasma insulin.[4] Testosterone, sex-hormone binding globulin and Vitamin D were measured by mass spectrometry. Informed written consent was obtained from all participants, and the research protocols were approved by the Ethics Committee of Northern Ostrobotnia Hospital District, Finland.

The Cardiovascular Risk in Young Finns Study (YFS) was designed to study associations of childhood risk factors to disease in adulthood (youngfinnsstudy.utu.fi).[5] The baseline study conducted in 1980 included n=3596 children and adolescents aged 3–18. Data in the cross-sectional analyses in the present study are from the 2001 survey, which included n=2283 participants (response rate 64%). These individuals were representative of the baseline cohort.[5] Among these, n=2247 individuals provided an overnight fasting blood samples, and the resulting serum samples were stored at -80°C prior to metabolic profiling by serum NMR metabolomics. After exclusion of individuals missing data on alcohol consumption (n=33) or alcohol intake in the highest percentile (n=13) as well as pregnant women (n=61) and subjects on lipid-lowering medication (n=7), n=2133 participants were included for the present analyses. Additional follow-up surveys were conducted in the years 2007 and 2011 with n=2204 and n=2080 participants, respectively. Longitudinal data on 6-year change in alcohol intake and change in metabolite concentrations were available for n=1466 individuals, who participated in both the 2001 and 2007 field studies.[4] As a sensitivity analysis, the resemblance

between longitudinal and cross-sectional association patterns was also assessed for 10-year follow-up (2001→2011) for n=1401 participants aged 34–49.[4,6] The questionnaires on alcohol intake were identical across the three adulthood field surveys. Physical activity index was assessed by metabolic-equivalent-of-task.[2] Current smoking was assessed by questionnaires. In addition to NMR metabolite profiling, the following plasma biomarkers were measured by standard clinical chemistry assays and analysed in the present study: C-reactive protein, alanine aminotransferase, gamma-glutamyl aminotransferase, leptin, adiponectin, vitamin D, and insulin.[4,5] Testosterone and sex-hormone binding globulin were measured by mass spectrometry for both women and men in the 2001 field study, but only for men in the 2007 field study.[4] All participants gave written informed consent, and the study was approved by the ethics committees of each of the five participating medical university study sites in Finland.

The FINRISK 1997 study was conducted in to monitor the health of the Finnish population among persons aged 24–74 at recruitment (thl.fi/finriski).[7,8] In total, n=8444 individuals were recruited during the spring of 1997 to represent the general population of the study areas. Serum NMR metabolic profiling was performed for n=7603 individuals (all individuals with sufficient serum sample available; 90% of the entire study population).[4] Only individuals under the age of 46 were included the analyses of the present study to minimize the influence of aging and reverse causality from comorbidities of high alcohol intake (n=4230 individuals aged 46–74 omitted). After exclusion of individuals who had missing data on alcohol consumption (n=473) or alcohol intake in the highest percentile (n=24) as well as pregnant women (n=58) and subjects on lipid-lowering medication (n=10), n=2808 participants were included for the present analyses. Participants filled in questionnaires of alcohol usage, smoking status, and physical activity. In the absence of sufficient information to derive metabolic-equivalent-of-task, a binary physical activity index was defined based on the self-reported level of leisure time physical activity.[4] Blood samples were stored at –70°C for later biomarker analyses. Samples were semi-fasting: participants were asked not to eat 4 hours prior to giving blood. The median fasting time was 5h (interquartile range 4–6h). In addition to NMR-based metabolite profiling, the following circulating biomarkers were assayed by standard clinical chemistry assays and analysed in the present study: C-reactive protein, gamma-glutamyl aminotransferase, adiponectin, testosterone, vitamin D, and insulin.[4,8] Participants gave written informed consent and the study was approved by the Coordinating Ethical Committee of the Helsinki and Uusimaa Hospital District, Finland.

References:

1. Järvelin MR, Sovio U, King V, et al. Early life factors and blood pressure at age 31 years in the 1966 northern Finland birth cohort. *Hypertension* 2004;44:838–46.
2. Würtz P, Mäkinen VP, Soininen P, et al. Metabolic signatures of insulin resistance in 7,098 young adults. *Diabetes* 2012;61:1372–1380.
3. Kujala UM, Mäkinen VP, Heinonen I, et al. Long-term leisure-time physical activity and serum metabolome. *Circulation* 2013;127:340-348.
4. Würtz P, Wang Q, Kangas AJ, et al. Metabolic signatures of adiposity in young adults: Mendelian randomization analysis and effects of weight change. *PLoS Med* 2014;11(12):e1001765.
5. Raitakari OT, Juonala M, Rönnemaa T, et al. Cohort profile: The cardiovascular risk in young Finns study. *Int J Epidemiol* 2008;37:1220–1226.
6. Nuotio J, Oikonen M, Magnussen CG, et al. Cardiovascular risk factors in 2011 and secular trends since 2007: the Cardiovascular Risk in Young Finns Study. *Scand J Public Health* 2014;42:563-71.
7. Vartiainen E, Laatikainen T, Peltonen M, et al. Thirty-five-year trends in cardiovascular risk factors in Finland. *Int J Epidemiol* 2010;39:504–518.
8. Blankenberg S, Zeller T, Saarela O, et al. Contribution of 30 biomarkers to 10-year cardiovascular risk estimation in 2 population cohorts: the MONICA, risk, genetics, archiving, and monograph (MORGAM) biomarker project. *Circulation* 2010;121:2388–2397.
9. Soininen P, Kangas AJ, Würtz P, Suna T, Ala-Korpela M. Quantitative serum nuclear magnetic resonance metabolomics in cardiovascular epidemiology and genetics. *Circ Cardiovasc Genet* 2015;8:192–206.
10. Kettunen J, Demirkan A, Würtz P, et al. Genome-wide study for circulating metabolites identifies 62 loci and reveals novel systemic effects of LPA. *Nat Commun* 2016;**7**:11122.

Table S1: Mean (SD) concentrations of metabolic measures in the study population, and association magnitudes in absolute concentration units.

Metabolic measure	Mean (SD)	Absolute concentration difference per 100 g/weekly ethanol intake<br>Beta [95%CI]; P-value (cross-sectional meta-analysis of 9778 individuals)	Absolute concentration change per 100 g change in weekly ethanol intake<br>Beta [95%CI]; P-value (longitudinal analysis of 1466 individuals)
**Lipoprotein lipid concentration**			
Extremely large VLDL [mmol/L]	0.020 (0.028)	0.0018 [0.0011, 0.0025] * $P=9\times10^{-7}$	0.00053 [-0.0012, 0.0022] * P=0.54
Very large VLDL [mmol/L]	0.053 (0.067)	0.0046 [0.0029, 0.0063] * $P=6\times10^{-8}$	0.0019 [-0.0023, 0.0061] * P=0.37
Large VLDL [mmol/L]	0.21 (0.23)	0.016 [0.011, 0.022] * $P=2\times10^{-9}$	0.013 [-0.00065, 0.027] * P=0.06
Medium VLDL [mmol/L]	0.47 (0.33)	0.017 [0.0096, 0.024] $P=6.5\times10^{-6}$	0.015 [-0.0037, 0.034] P=0.12
Small VLDL [mmol/L]	0.63 (0.25)	0.0013 [-0.0049, 0.0074] P=0.69	-0.0023 [-0.016, 0.011] P=0.74
Very small VLDL [mmol/L]	0.53 (0.17)	-0.0043 [-0.0087, 0.000032] P=0.05	-0.0044 [-0.013, 0.0043] P=0.32
IDL [mmol/L]	1.21 (0.33)	-0.017 [-0.026, -0.0084] P=0.0001	-0.012 [-0.029, 0.0049] P=0.16
Large LDL [mmol/L]	1.50 (0.43)	-0.018 [-0.028, -0.0067] P=0.001	-0.013 [-0.035, 0.0086] P=0.24
Medium LDL [mmol/L]	0.89 (0.27)	-0.0061 [-0.013, 0.00065] P=0.08	-0.0049 [-0.019, 0.0086] P=0.47
Small LDL [mmol/L]	0.57 (0.18)	0.0016 [-0.0030, 0.0062] P=0.50	0.0016 [-0.0075, 0.011] P=0.74
Very large HDL [mmol/L]	0.52 (0.27)	0.018 [0.012, 0.024] $P=1\times10^{-9}$	0.0086 [-0.0036, 0.021] P=0.17
Large HDL [mmol/L]	0.86 (0.40)	0.050 [0.040, 0.059] $P=9\times10^{-25}$	0.027 [0.0063, 0.047] P=0.01
Medium HDL [mmol/L]	0.90 (0.28)	0.058 [0.051, 0.064] $P=2\times10^{-72}$	0.036 [0.018, 0.055] $P=8\times10^{-5}$
Small HDL [mmol/L]	1.16 (0.20)	0.033 [0.028, 0.037] $P=7\times10^{-53}$	0.022 [0.010, 0.034] P=0.0003
**Lipoprotein particle size**			
VLDL particle diameter [nm]	36.1 (1.38)	0.13 [0.098, 0.16] $P=4\times10^{-15}$	0.12 [0.038, 0.21] P=0.005
LDL particle diameter [nm]	23.5 (0.17)	-0.027 [-0.032, -0.023] $P=3\times10^{-32}$	-0.016 [-0.027, -0.0042] P=0.007
HDL particle diameter [nm]	9.98 (0.28)	0.018 [0.011, 0.024] $P=3\times10^{-8}$	0.0084 [-0.0032, 0.020] P=0.16
**Apolipoproteins**			
Apolipoprotein B [g/L]	0.96 (0.25)	-0.0071 [-0.014, -0.00076] P=0.03	-0.0057 [-0.018, 0.0070] P=0.38
Apolipoprotein A-I [g/L]	1.69 (0.24)	0.048 [0.042, 0.055] $P=6\times10^{-50}$	0.034 [0.019, 0.050] $P=2\times10^{-5}$
Apolipoprotein B/ apolipoprotein A-I	0.58 (0.15)	-0.020 [-0.024, -0.016] $P=1\times10^{-25}$	-0.015 [-0.022, -0.0078] $P=5\times10^{-5}$
**Cholesterol (C)**			
Total C [mmol/L]	5.19 (1.12)	0.027 [-0.00080, 0.056] P=0.06	-0.0036 [-0.068, 0.061] P=0.91

Metabolic measure	Mean (SD)	Absolute concentration difference per 100 g/weekly ethanol intake<br>Beta [95%CI]; P-value (cross-sectional meta-analysis of 9778 individuals)	Absolute concentration change per 100 g change in weekly ethanol intake<br>Beta [95%CI]; P-value (longitudinal analysis of 1466 individuals)
Non-HDL C [mmol/L]	3.54 (1.04)	-0.0451 [-0.071, -0.019] P=0.0007	-0.0457 [-0.099, 0.0070] P=0.09
Remnant C [mmol/L]	1.52 (0.45)	-0.0193 [-0.0301, -0.0079] P=0.0009	-0.025 [-0.0503, 0.00036] P=0.05
VLDL C [mmol/L]	0.74 (0.28)	-0.00446 [-0.012, 0.0026] P=0.21	-0.0116 [-0.0285, 0.0052] P=0.18
IDL C [mmol/L]	0.78 (0.21)	-0.0149 [-0.020, -0.0095] $P=7\times10^{-8}$	-0.0133 [-0.0247, -0.0019] P=0.02
LDL C [mmol/L]	2.02 (0.63)	-0.0243 [-0.040, -0.0085] P=0.003	-0.0208 [-0.0529, 0.011] P=0.21
HDL C [mmol/L]	1.65 (0.40)	0.0733 [0.064, 0.083] $P=3\times10^{-50}$	0.0421 [0.0193, 0.065] P=0.0003
HDL_2 C [mmol/L]	1.12 (0.40)	0.0673 [0.058, 0.077] $P=6\times10^{-42}$	0.0366 [0.0134, 0.060] P=0.002
HDL_3 C [mmol/L]	0.52 (0.049)	0.00587 [0.0046, 0.0072] $P=2\times10^{-19}$	0.00551 [0.00196, 0.0090] P=0.002
**Triglycerides**			
Total triglycerides [mmol/L]	1.18 (0.59)	0.041 [0.026, 0.056] $P=6\times10^{-8}$	0.0277 [-0.00504, 0.060] P=0.10
VLDL TG [mmol/L]	0.67 (0.48)	0.0334 [0.022, 0.045] $P=4\times10^{-9}$	0.0262 [-0.00128, 0.054] P=0.06
LDL TG [mmol/L]	0.27 (0.11)	0.00138 [-0.0012, 0.0039] P=0.29	-0.00208 [-0.00712, 0.0029] P=0.42
HDL TG [mmol/L]	0.010 (0.051)	0.00255 [0.0013, 0.0038] $P=8\times10^{-5}$	0.00274 [-0.00133, 0.0068] P=0.19
**Phospholipids**			
Total phospholipids [mmol/L]	3.10 (0.59)	0.085 [0.071, 0.099] $P=1\times10^{-30}$	0.0559 [0.0175, 0.094] P=0.004
VLDL PL [mmol/L]	0.44 (0.20)	0.00624 [0.0013, 0.011] P=0.01	0.0032 [-0.00728, 0.014] P=0.55
LDL PL [mmol/L]	0.66 (0.19)	0.00207 [-0.0025, 0.0067] P=0.38	0.0064 [-0.00445, 0.017] P=0.25
HDL PL [mmol/L]	1.68 (0.40)	0.0822 [0.073, 0.092] $P=3\times10^{-64}$	0.0487 [0.0248, 0.073] $P=7\times10^{-5}$
Phosphoglycerides [mmol/L]	1.72 (0.46)	0.080 [0.069, 0.091] $P=2\times10^{-43}$	0.055 [0.026, 0.085] P=0.0003
Cholines [mmol/L]	2.04 (0.49)	0.0844 [0.072, 0.097] $P=4\times10^{-42}$	0.0516 [0.0161, 0.087] P=0.005
Sphingomyelin [mmol/L]	0.32 (0.085)	-0.00224 [-0.0041, -0.00038] P=0.02	-0.00397 [-0.00942, 0.0015] P=0.15
**Fatty acids**			
Total fatty acids [mmol/L]	11.4 (3.13)	0.336 [0.26, 0.42] $P=6\times10^{-16}$	0.187 [0.00433, 0.37] P=0.05
Saturated fatty acids [mmol/L]	3.79 (1.17)	0.0971 [0.066, 0.13] $P=6\times10^{-10}$	0.066 [-0.00627, 0.14] P=0.07
Monounsaturated fatty acids (MUFA) [mmol/L]	3.30 (1.18)	0.213 [0.18, 0.24] $P=1\times10^{-41}$	0.136 [0.0633, 0.21] P=0.0003
Polyunsaturated fatty acids (PUFA) [mmol/L]	4.29 (0.98)	0.0292 [0.0042, 0.054] P=0.02	-0.0161 [-0.071, 0.039] P=0.57

Metabolic measure	Mean (SD)	Absolute concentration difference per 100 g/weekly ethanol intake<br>Beta [95%CI]; P-value (cross-sectional meta-analysis of 9778 individuals)	Absolute concentration change per 100 g change in weekly ethanol intake<br>Beta [95%CI]; P-value (longitudinal analysis of 1466 individuals)
Omega-6 fatty acids [mmol/L]	3.88 (0.89)	0.0115 [-0.0111, 0.034]<br>P=0.32	-0.0182 [-0.0677, 0.0312]<br>P=0.47
Linoleic acid [mmol/L]	3.28 (0.73)	-0.0177 [-0.0367, 0.00117]<br>P=0.07	-0.028 [-0.0704, 0.0145]<br>P=0.20
Omega-3 fatty acids [mmol/L]	0.41 (0.15)	0.0177 [0.0137, 0.0218]<br>$P=7\times10^{-18}$	0.00201 [-0.00822, 0.0122]<br>P=0.70
Docosahexaenoic acid (DHA) [mmol/L]	0.17 (0.072)	0.00806 [0.00613, 0.00999]<br>$P=3\times10^{-16}$	-0.00133 [-0.00642, 0.00375]<br>P=0.61
**Fatty acid ratios to total fatty acids**			
Saturated fatty acids [%]	33.1 (2.66)	-0.101 [-0.171, -0.0299]<br>P=0.005	0.0948 [-0.109, 0.299]<br>P=0.36
Monounsaturated fatty acids (MUFA) [%]	28.6 (3.67)	0.903 [0.81, 0.997]<br>$P=1\times10^{-79}$	0.68 [0.44, 0.92]<br>$P=4\times10^{-8}$
Polyunsaturated fatty acids (PUFA) [%]	38.2 (3.90)	-0.819 [-0.922, -0.715]<br>$P=4\times10^{-54}$	-0.772 [-1.07, -0.477]<br>$P=3.3\times10^{-7}$
Omega-6 fatty acids [%]	34.6 (3.85)	-0.87 [-0.972, -0.768]<br>$P=2\times10^{-62}$	-0.73 [-1.02, -0.44]<br>$P=1\times10^{-6}$
Linoleic acid [%]	29.3 (3.92)	-0.937 [-1.04, -0.832]<br>$P=9\times10^{-69}$	-0.724 [-0.995, -0.454]<br>$P=2\times10^{-7}$
Omega-3 fatty acids [%]	3.62 (0.99)	0.0538 [0.0274, 0.0803]<br>$P=6\times10^{-5}$	-0.0433 [-0.118, 0.0309]<br>P=0.25
Docosahexaenoic acid (DHA) [%]	1.57 (0.55)	0.0274 [0.0128, 0.0421]<br>P=0.0003	-0.0328 [-0.0712, 0.00564]<br>P=0.10
Unsaturation degree	1.26 (0.083)	-0.00567 [-0.00793, -0.00342]<br>$P=8\times^{10-7}$	-0.0119 [-0.0181, -0.00584]<br>P=0.0001
**Amino acids**			
Glutamine [µmol/L]	524 (96.7)	-10.1 [-12.2, -7.91]<br>$P=5\times10^{-20}$	-7.15 [-14, -0.287]<br>P=0.04
Glycine [µmol/L]	322 (68.8)	-1.92 [-3.69, -0.141]<br>P=0.03	-6.49 [-10.7, -2.31]<br>P=0.002
Alanine [µmol/L]	429 (73.9)	3.06 [1.18, 4.94]<br>P=0.002	3.13 [-2.24, 8.5]<br>P=0.25
Histidine [µmol/L]	69.2 (12.5)	0.387 [0.0778, 0.697]<br>P=0.01	-0.561 [-1.67, 0.547]<br>P=0.32
Isoleucine [µmol/L]	56.4 (17.7)	0.418 [-0.0128, 0.849]<br>P=0.06	1.1 [0.103, 2.11]<br>P=0.031
Leucine [µmol/L]	89.0 (21.3)	0.873 [0.363, 1.38]<br>P=0.0008	0.925 [-0.335, 2.19]<br>P=0.15
Valine [µmol/L]	215 (48)	-1.35 [-2.5, -0.205]<br>P=0.02	-2.24 [-5.24, 0.751]<br>P=0.14
Phenylalanine [µmol/L]	82.0 (14.4)	0.602 [0.239, 0.964]<br>P=0.001	-0.414 [-1.42, 0.595]<br>P=0.42
Tyrosine [µmol/L]	53.2 (13.1)	1.09 [0.762, 1.42]<br>$P=9\times10^{-11}$	-0.152 [-1.02, 0.72]<br>P=0.73
**Glycolysis and gluconeogenesis metabolites**			
Glucose [mmol/L]	4.75 (1.0)	0.0283 [0.00551, 0.0511] *<br>P=0.02	0.00156 [-0.0547, 0.0578] *<br>P=0.96
Lactate [µmol/L]	1.47 (0.44)	0.00531 [-0.00473, 0.0154]<br>P=0.30	0.00153 [-0.0307, 0.0338]<br>P=0.93

Metabolic measure	Mean (SD)	Absolute concentration difference per 100 g/weekly ethanol intake<br>Beta [95%CI]; P-value (cross-sectional meta-analysis of 9778 individuals)	Absolute concentration change per 100 g change in weekly ethanol intake<br>Beta [95%CI]; P-value (longitudinal analysis of 1466 individuals)
Pyruvate [µmol/L]	83.7 (26.9)	1.43 [0.767, 2.1] $P=3\times10^{-5}$	0.614 [-1.67, 2.9] P=0.60
Glycerol [µmol/L]	99.9 (46.2)	2.77 [1.68, 3.85] $P=6\times10^{-7}$	2.41 [-0.606, 5.43] P=0.12
**Ketone bodies**			
Acetoacetate [µmol/L]	59.9 (45.1)	2.62 [1.47, 3.77] * $P=8\times10^{-6}$	-0.879 [-3.99, 2.23] * P=0.58
Beta-hydroxybutyrate [µmol/L]	107 (40)	-0.263 [-1.41, 0.883] P=0.65	-1.27 [-4.79, 2.26] P=0.48
**Miscellaneous**			
Citrate [µmol/L]	107 (20)	-3.35 [-3.87, -2.84] $P=9\times10^{-38}$	-2.54 [-4.23, -0.856] P=0.003
Creatinine [µmol/L]	62.9 (13.5)	-0.0159 [-0.309, 0.277] P=0.92	-0.61 [-1.31, 0.0865] P=0.09
Albumin [cu]	102 (14)	0.156 [-0.0898, 0.402] P=0.21	-0.463 [-1.51, 0.581] P=0.39
Acetate [µmol/L]	44.8 (13.2)	-0.524 [-0.868, -0.181] P=0.003	-0.354 [-1.48, 0.768] P=0.54
**Inflammatory markers**			
Glycoprotein acetyls (GlycA) [mmol/L]	1.35 (0.25)	0.0166 [0.0102, 0.023] $P=3\times10^{-7}$	0.00715 [-0.0111, 0.0254] P=0.44
C-reactive protein [mg/L]	1.81 (3.32)	0.152 [0.0665, 0.238] * P=0.0005	0.116 [-0.0951, 0.328] * P=0.28
Phospholipase activity [cu]	9.92 (0.99)	-0.0279 [-0.0621, 0.00623] P=0.11	NA
**Hormones**			
Testosterone (Men) [nmol/L]	20.1 (6.81)	0.536 [0.324, 0.747] $P=7\times10^{-7}$	0.62 [0.149, 1.09] P=0.01
Testosterone (Women) [nmol/L]	1.61 (0.79)	0.0741 [0.0372, 0.111] $P=8\times10^{-5}$	NA
Sex-hormone binding globulin (Men) [nmol/L]	32.6 (12.9)	-0.864 [-1.35, -0.378] P=0.0005	-0.639 [-1.22, -0.0596] P=0.03
Sex-hormone binding globulin (Women) [nmol/L]	69.2 (42.3)	1.98 [-0.615, 4.57] P=0.13	NA
Vitamin-D [cu]	2.15 (1.03)	0.00794 [-0.0196, 0.0355] P=0.57	-0.0499 [-0.117, 0.0171] P=0.14
Leptin [ng/mL]	10.4 (9.54)	0.019 [-0.276, 0.314] P=0.90	NA
Insulin [IU/L]	7.41 (4.35)	-0.0814 [-0.195, 0.0326] * P=0.16	0.0486 [-0.326, 0.423] * P=0.8
Adiponectin [µg/mL]	7.74 (4.40)	0.373 [0.235, 0.512] $P=1.3\times10^{-7}$	0.317 [0.0802, 0.555] P=0.009
**Liver function markers**			
Gamma-glutamine aminotransferase [cu]	9.94 (0.57)	0.122 [0.108, 0.136] * $P=2\times10^{-68}$	0.079 [0.0466, 0.111] * $P=2\times10^{-6}$
Alanine amino transferase [cu]	9.97 (0.91)	0.0544 [0.0228, 0.086] P=0.0007	0.0279 [-0.027, 0.0828] P=0.32

Mean concentrations of the metabolic measures and standard deviations (SD) were averaged across the three cohorts. Associations of alcohol intake with metabolic measures in absolute concentration units correspond to the results shown in the left columns of Figures 1–3 multiplied by the SD. The longitudinal associations of change in alcohol intake with change in lipid and metabolite concentrations correspond to the results of the right columns in Figures 1–3 multiplied by the SD. Several of the metabolic measures displayed non-linear associations with alcohol intake; the association magnitude in different ranges of alcohol consumption can be estimated from the slopes for the continuous association plots shown in Supplementary Figure 5.

*: Metabolic measures were log-transformed for the primary analyses, but results are shown here in non-transformed absolute concentration units to facilitate interpretation of association magnitudes.

The 76 metabolic measures listed first in the table were all quantified using the same single high-throughput serum NMR metabolomics platform for the three cohorts. [9] The 14 lipoprotein subclass sizes were defined as follows: extremely large VLDL with particle diameters from 75 nm upwards and a possible contribution of chylomicrons, five VLDL subclasses (average particle diameters of 64.0 nm, 53.6 nm, 44.5 nm, 36.8 nm, and 31.3 nm), IDL (28.6 nm), three LDL subclasses (25.5 nm, 23.0 nm, and 18.7 nm), and four HDL subclasses (14.3 nm, 12.1 nm, 10.9 nm, and 8.7 nm). The mean size for VLDL, LDL and HDL particles was calculated by weighting the corresponding subclass diameters with their particle concentrations. Remnant cholesterol was defined as VLDL-cholesterol + IDL-cholesterol. Correlation coefficients between the metabolic measures have been published previously.[4] Ten additional metabolic measures, listed in the bottom of the table, were assayed by standard clinical chemistry and mass-spectrometry in at least two of the cohorts.[4]

Coefficients of variation (CV) are below 5% for more than 75% of the metabolic measures, as assessed over thousands of samples.[10] Representative CVs are: total cholesterol 2.1%, LDL cholesterol 2.3%, HDL cholesterol 2.3%, total triglycerides 1.2%, apolipoprotein B 2.2%, apolipoprotein A-I 1.6%, omega-3 fatty acids 2.7%, omega-6 fatty acids 4.5%, proportion of omega-6 fatty acids relative to total fatty acids 2.2%, glucose 2.6%, leucine 1.9%, phenylalanine 3.9%, creatinine 3.9%, glycoprotein acetylation (GlycA) 1.1%. The CVs for lipoprotein subclass measures are generally below 5%.

Abbreviations: VLDL, very-low-density lipoprotein. LDL, low-density lipoprotein; HDL, high-density lipoprotein; cu = standardized concentration units.

Figure S1: Distributions of alcohol intake in the three population-based cohorts.

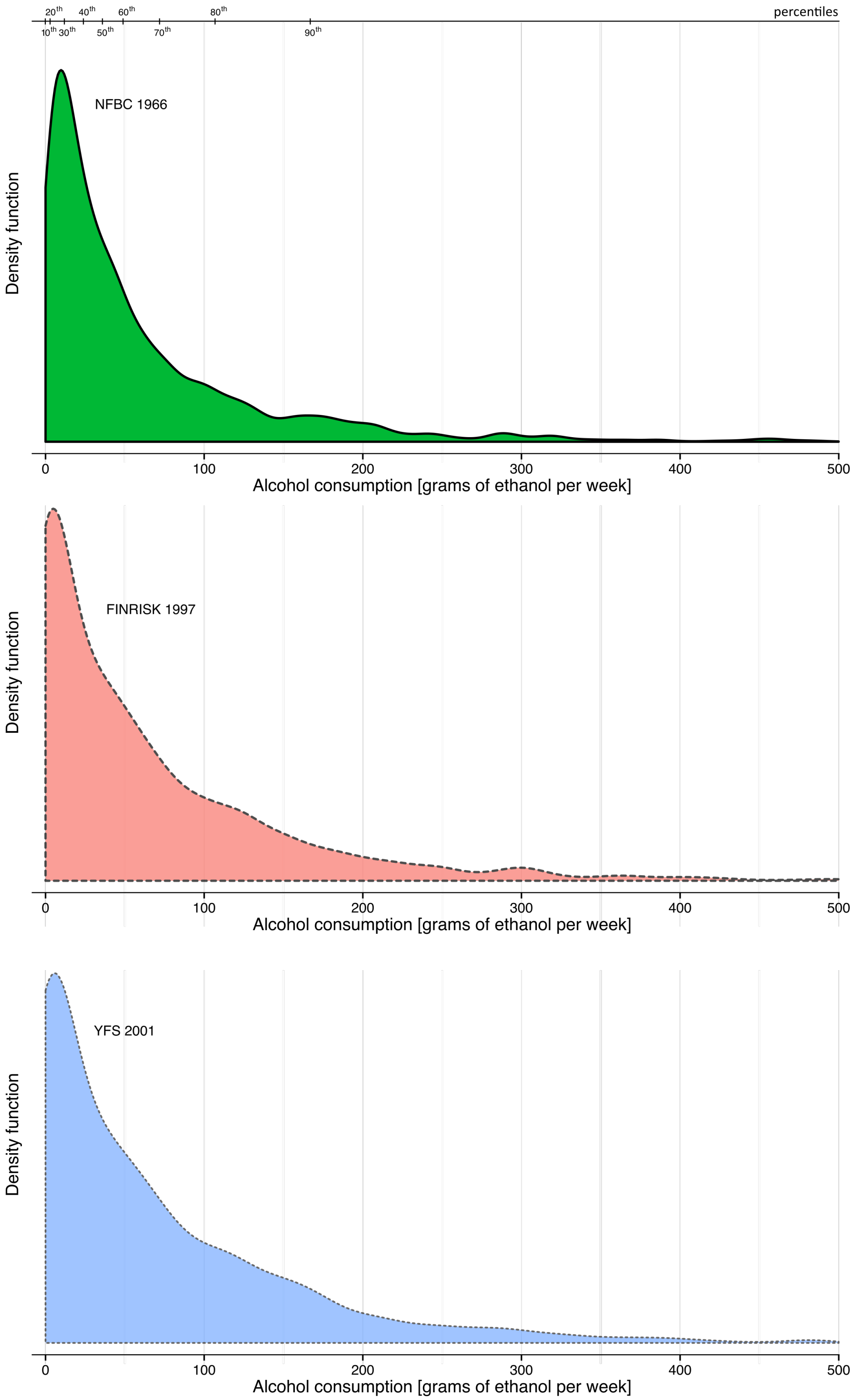

Density plots of the distributions of alcohol intake in the three cohorts of young adults. The information on alcohol intake was based on usual volume of ethanol consumed in the NFBC-1966 and on volume of ethanol consumed in the past week in the FINRISK 1997 study and in the YFS. The top axis indicates the percentiles of alcohol consumption in the total study population.

Figure S2: Lipid and metabolite concentrations as a function of alcohol intake.

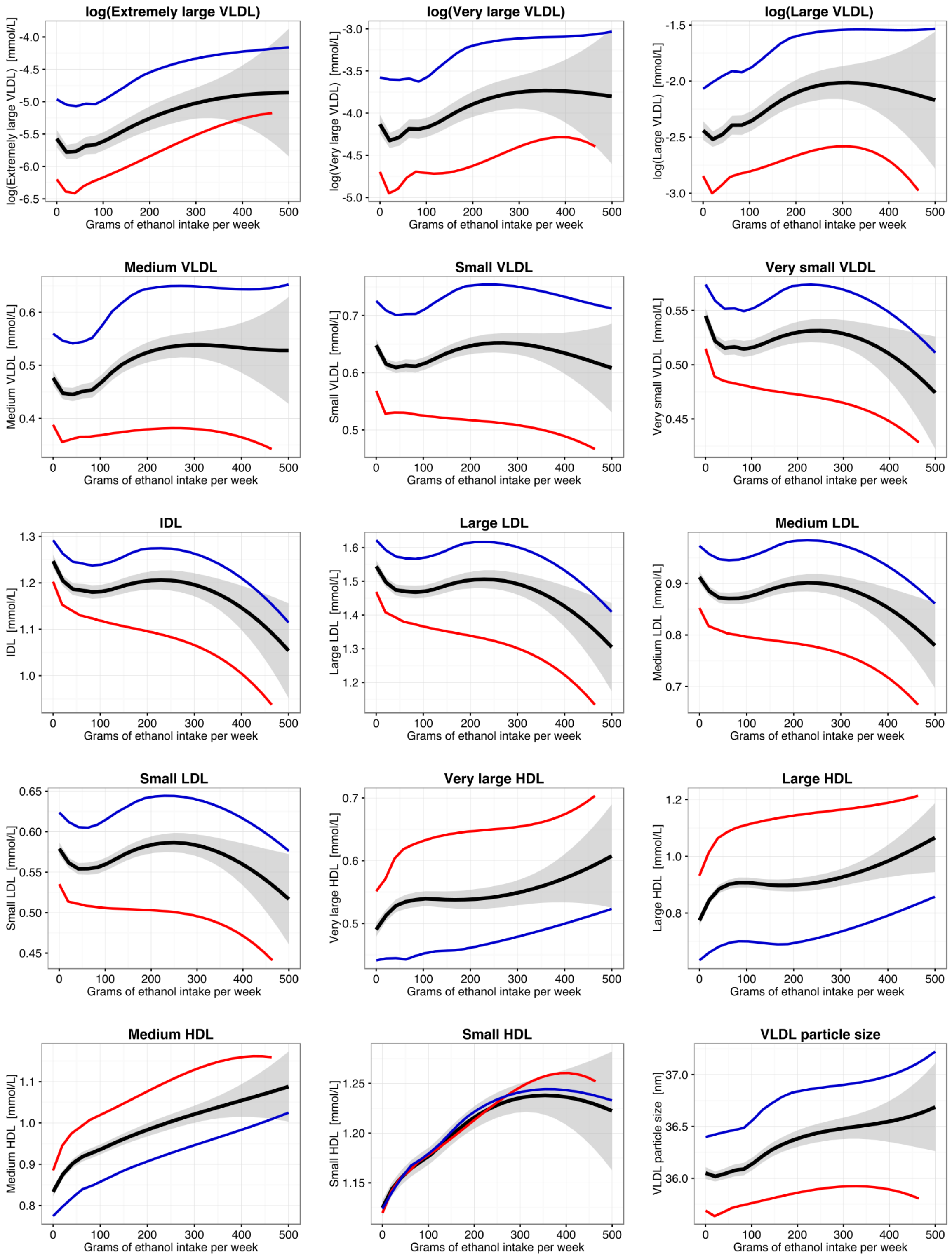

Continuous shape of the association between alcohol intake and 86 metabolic measures for men (blue; n=4707) and women (red; n=5071). The black curves denote the shape of the association for men and women combined, with the grey shaded area denoting the 95% confidence interval of the fit. The association shapes were derived using local quadratic regression fitting evaluated at 25 points. The metabolic measures were first adjusted for age and sex in each cohort, and then pooled by combining the residuals and scaling back to absolute units. Equivalent analyses were done stratified by sex. Similar trends were observed in each of the three cohorts.

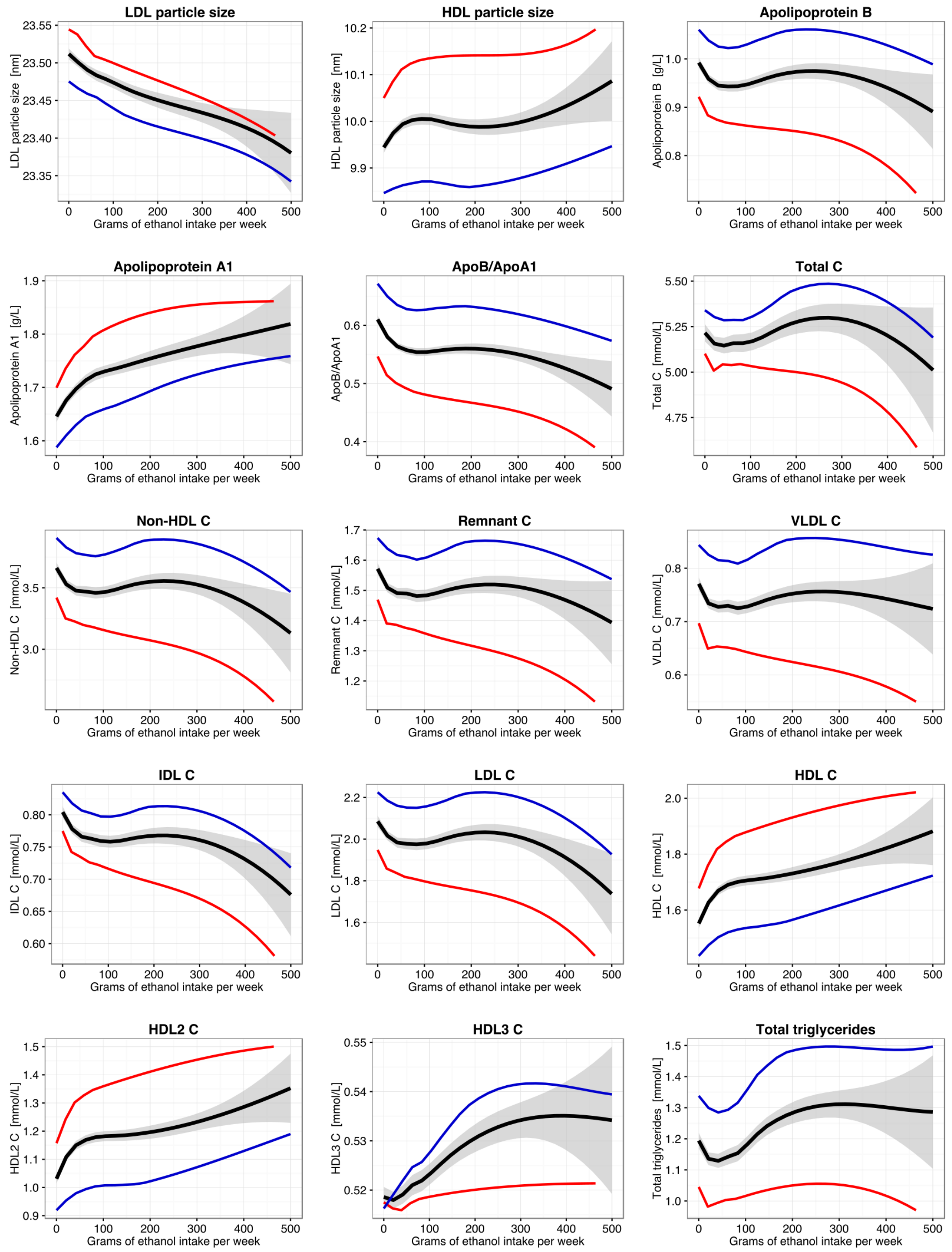

Continuous shape of the association between alcohol intake and 86 metabolic measures for men (blue; n=4707) and women (red; n=5071). The black curves denote the shape of the association for men and women combined, with the grey shaded area denoting the 95% confidence interval of the fit. The association shapes were derived using local quadratic regression fitting evaluated at 25 points. The metabolic measures were first adjusted for age and sex in each cohort, and then pooled by combining the residuals and scaling back to absolute units. Equivalent analyses were done stratified by sex. Similar trends were observed in each of the three cohorts.

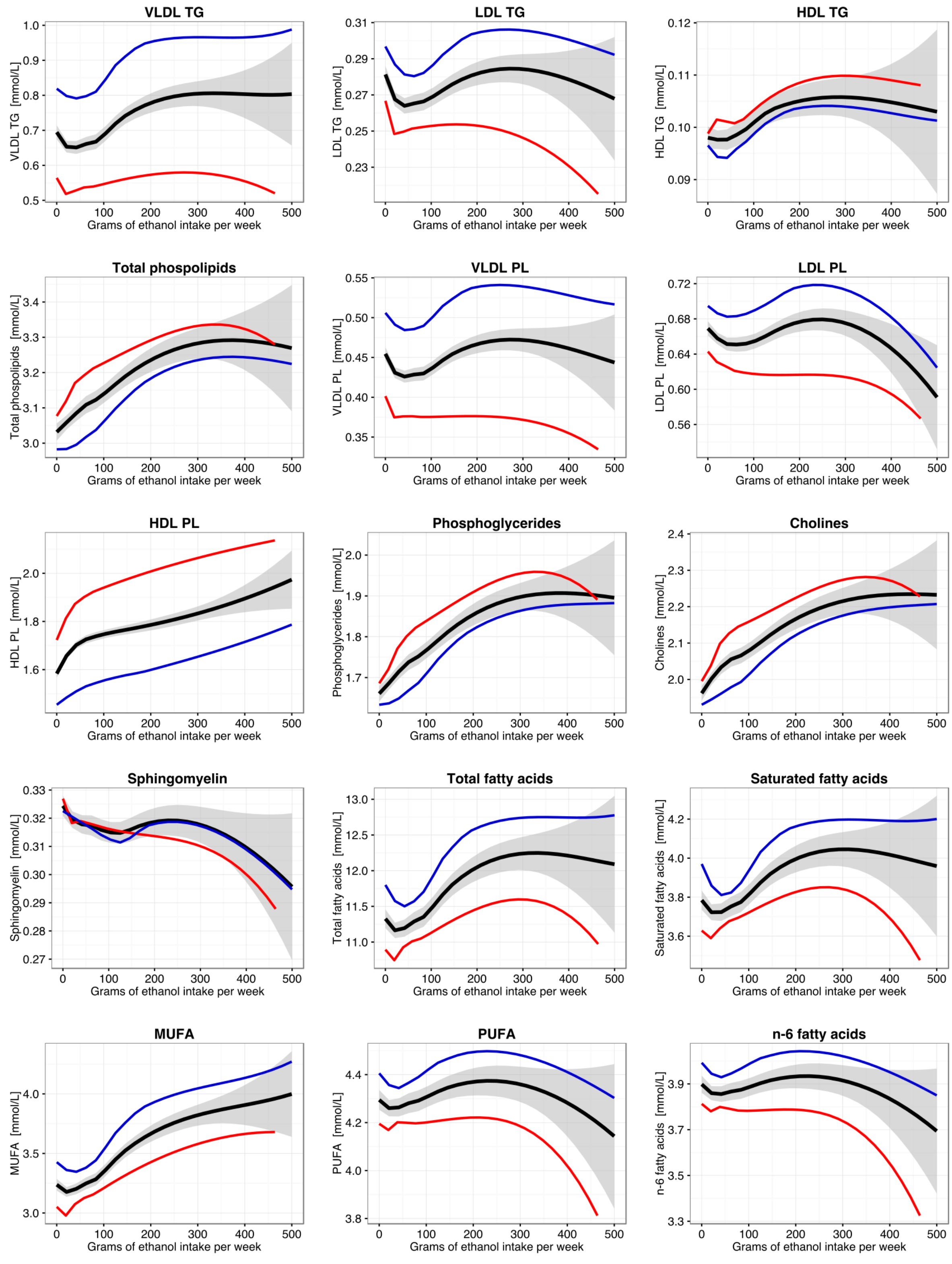

Continuous shape of the association between alcohol intake and 86 metabolic measures for men (blue; n=4707) and women (red; n=5071). The black curves denote the shape of the association for men and women combined, with the grey shaded area denoting the 95% confidence interval of the fit. The association shapes were derived using local quadratic regression fitting evaluated at 25 points. The metabolic measures were first adjusted for age and sex in each cohort, and then pooled by combining the residuals and scaling back to absolute units. Equivalent analyses were done stratified by sex. Similar trends were observed in each of the three cohorts.

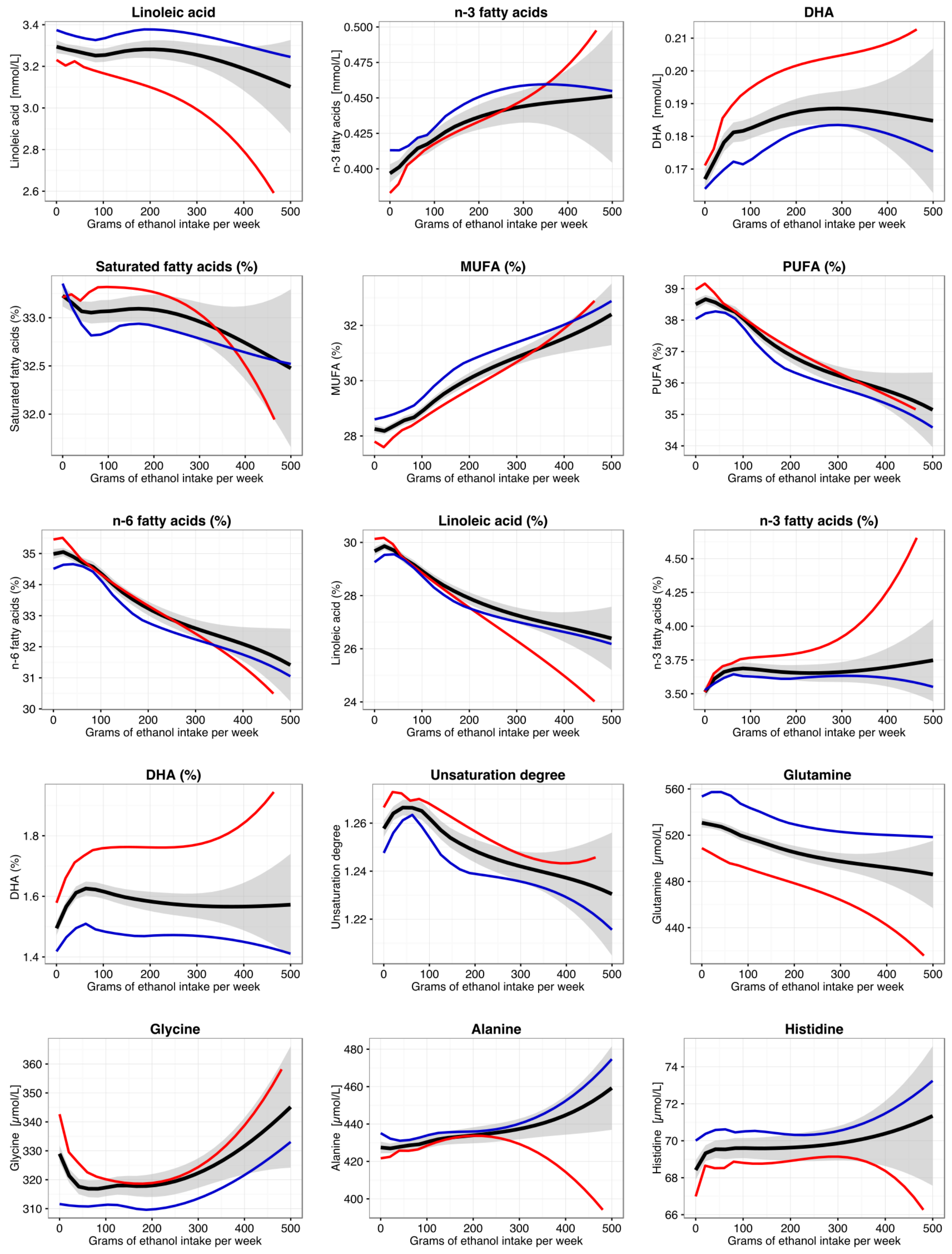

Continuous shape of the association between alcohol intake and 86 metabolic measures for men (blue; n=4707) and women (red; n=5071). The black curves denote the shape of the association for men and women combined, with the grey shaded area denoting the 95% confidence interval of the fit. The association shapes were derived using local quadratic regression fitting evaluated at 25 points. The metabolic measures were first adjusted for age and sex in each cohort, and then pooled by combining the residuals and scaling back to absolute units. Equivalent analyses were done stratified by sex. Similar trends were observed in each of the three cohorts.

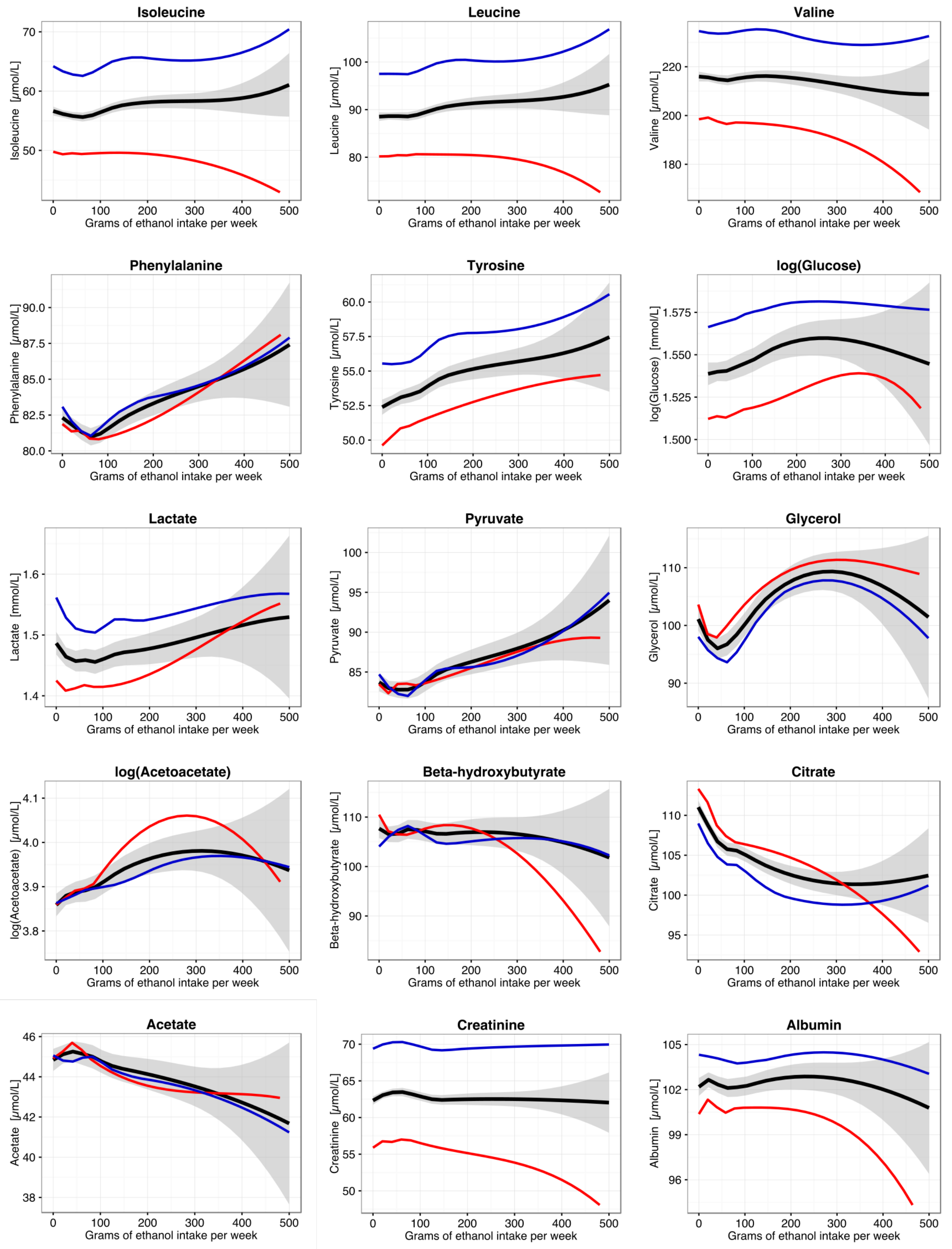

Continuous shape of the association between alcohol intake and 86 metabolic measures for men (blue; n=4707) and women (red; n=5071). The black curves denote the shape of the association for men and women combined, with the grey shaded area denoting the 95% confidence interval of the fit. The association shapes were derived using local quadratic regression fitting evaluated at 25 points. The metabolic measures were first adjusted for age and sex in each cohort, and then pooled by combining the residuals and scaling back to absolute units. Equivalent analyses were done stratified by sex. Similar trends were observed in each of the three cohorts.

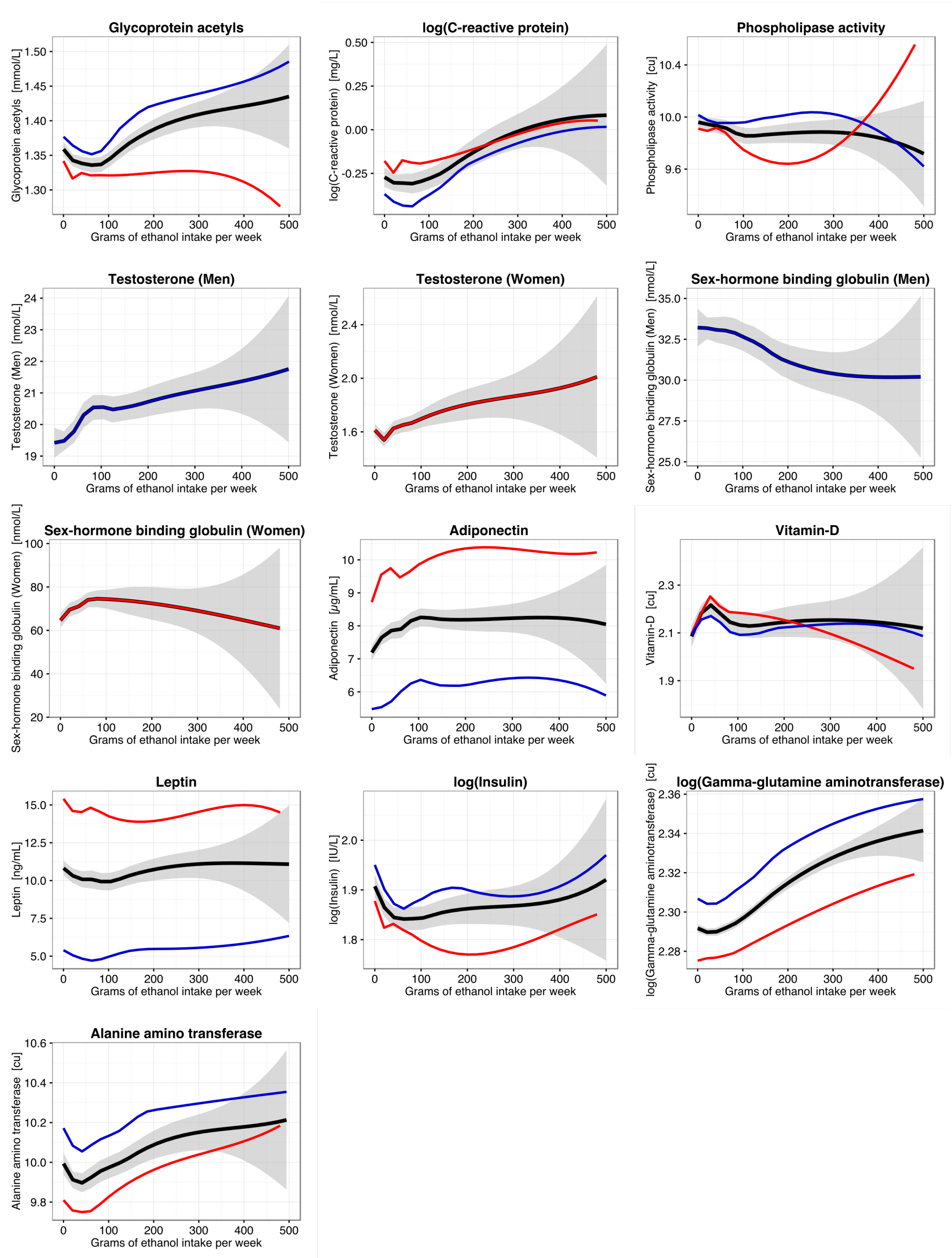

Continuous shape of the association between alcohol intake and 86 metabolic measures for men (blue; n=4707) and women (red; n=5071). The black curves denote the shape of the association for men and women combined, with the grey shaded area denoting the 95% confidence interval of the fit. The association shapes were derived using local quadratic regression fitting evaluated at 25 points. The metabolic measures were first adjusted for age and sex in each cohort, and then pooled by combining the residuals and scaling back to absolute units. Equivalent analyses were done stratified by sex. Similar trends were observed in each of the three cohorts.

Figure S3A. Cross-sectional associations between alcohol consumption and metabolic measures separately in the three population-based cohorts.

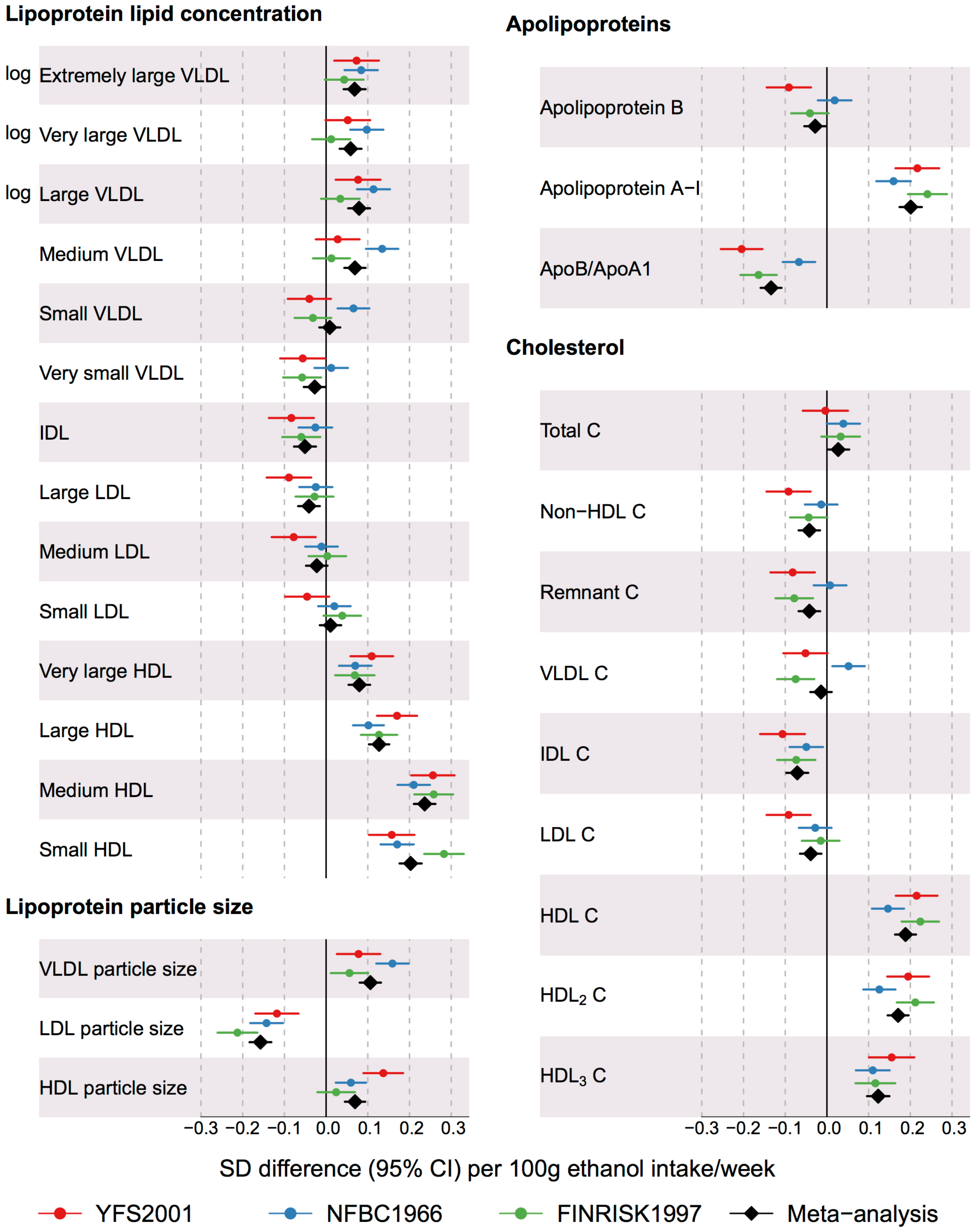

Cross-sectional associations of alcohol intake with lipoprotein lipids in the individual cohorts and meta-analysis (black diamonds; n=9778). The associations were adjusted for age and sex.

Figure S3B. Cross-sectional associations between alcohol consumption and metabolic measures separately in the three population-based cohorts.

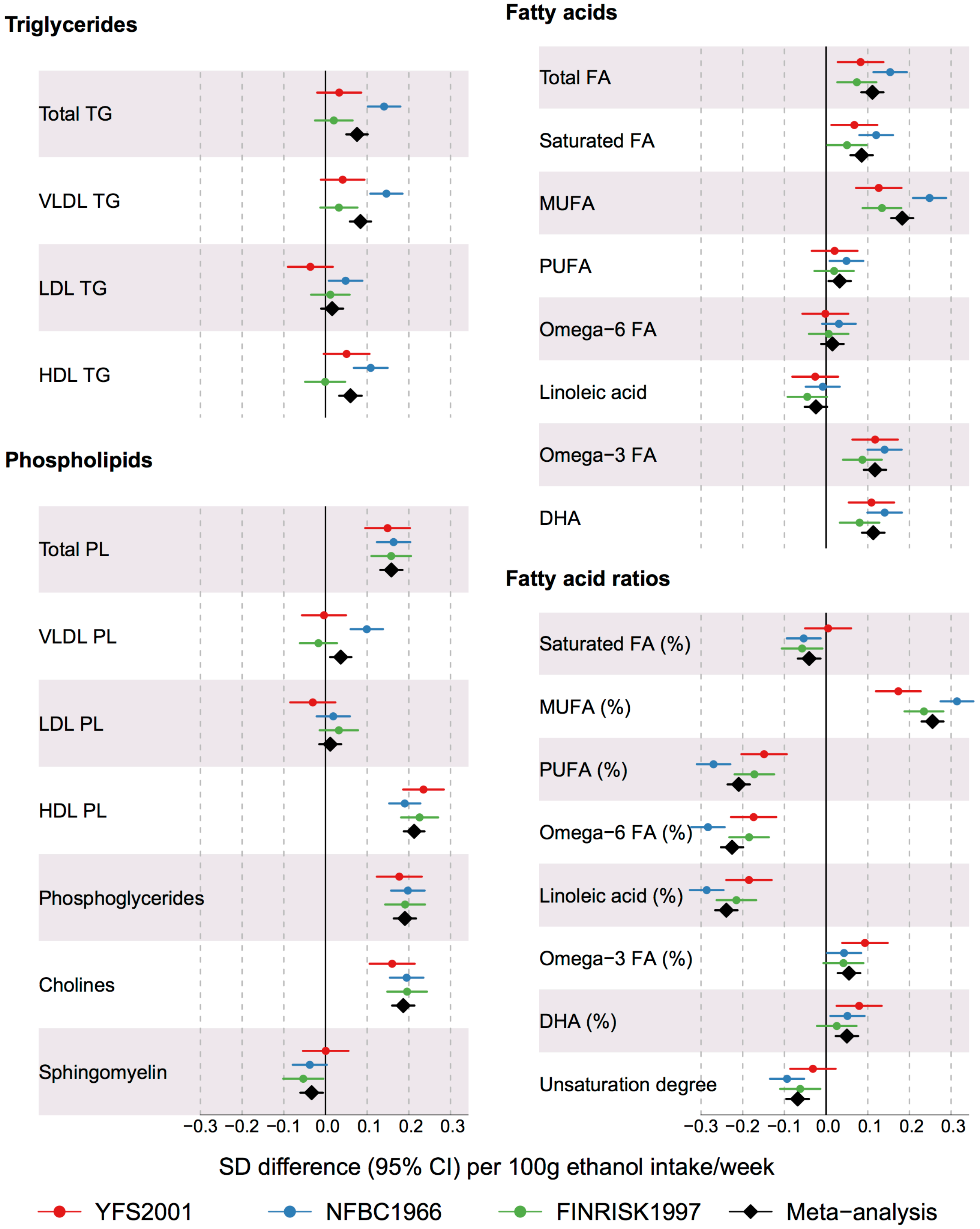

Cross-sectional associations of alcohol intake with fatty acid measures in the individual cohorts and meta-analysis (black diamonds; n=9778). The associations were adjusted for age and sex.

Figure S3C. Cross-sectional associations between alcohol consumption and metabolic measures separately in the three population-based cohorts.

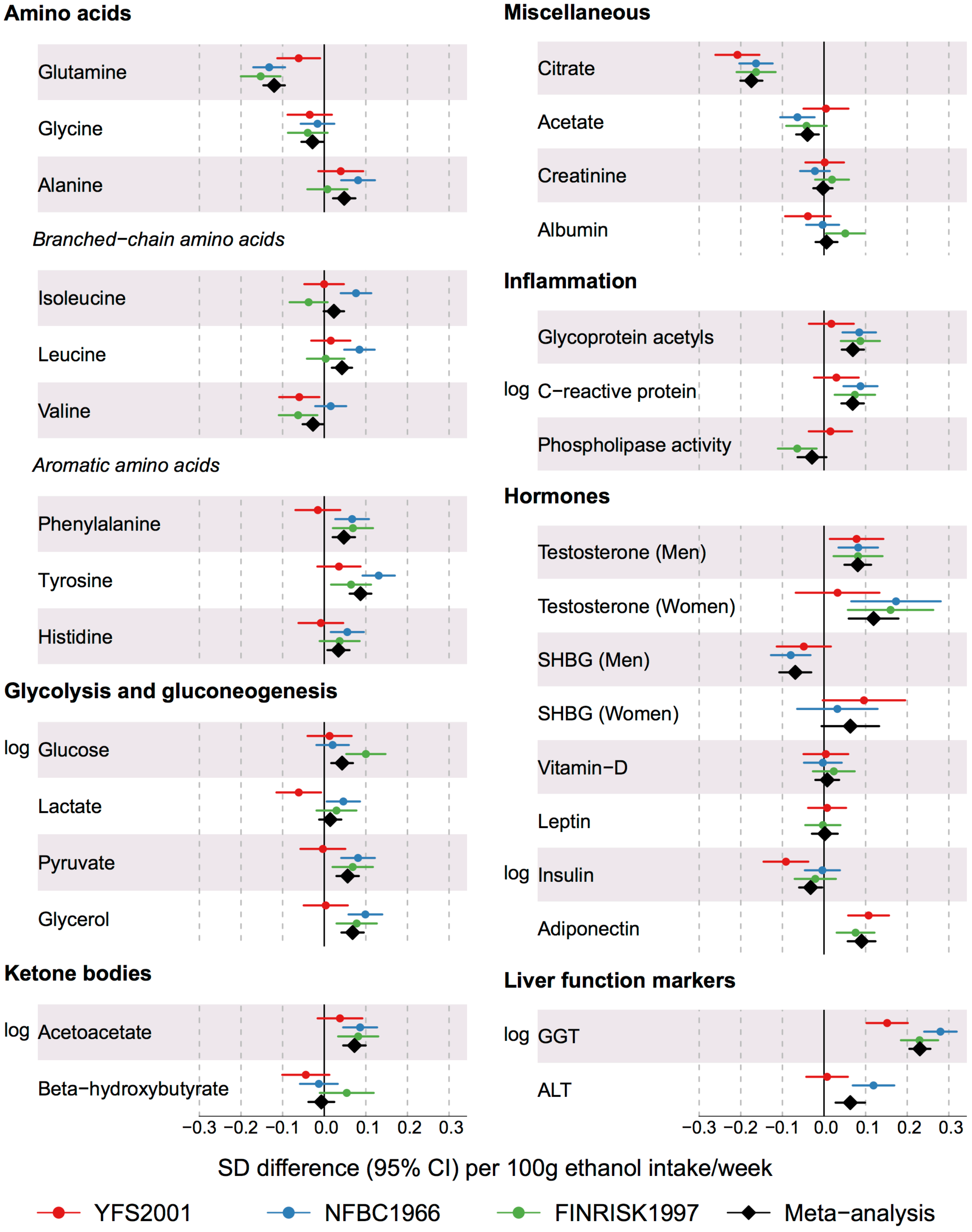

Cross-sectional associations of alcohol intake with metabolite and hormonal measures in the individual cohorts and meta-analysis (black diamonds; n=9778). The associations were adjusted for age and sex.

Figure S4. Cross-sectional associations between alcohol consumption and metabolic measures for men and women.

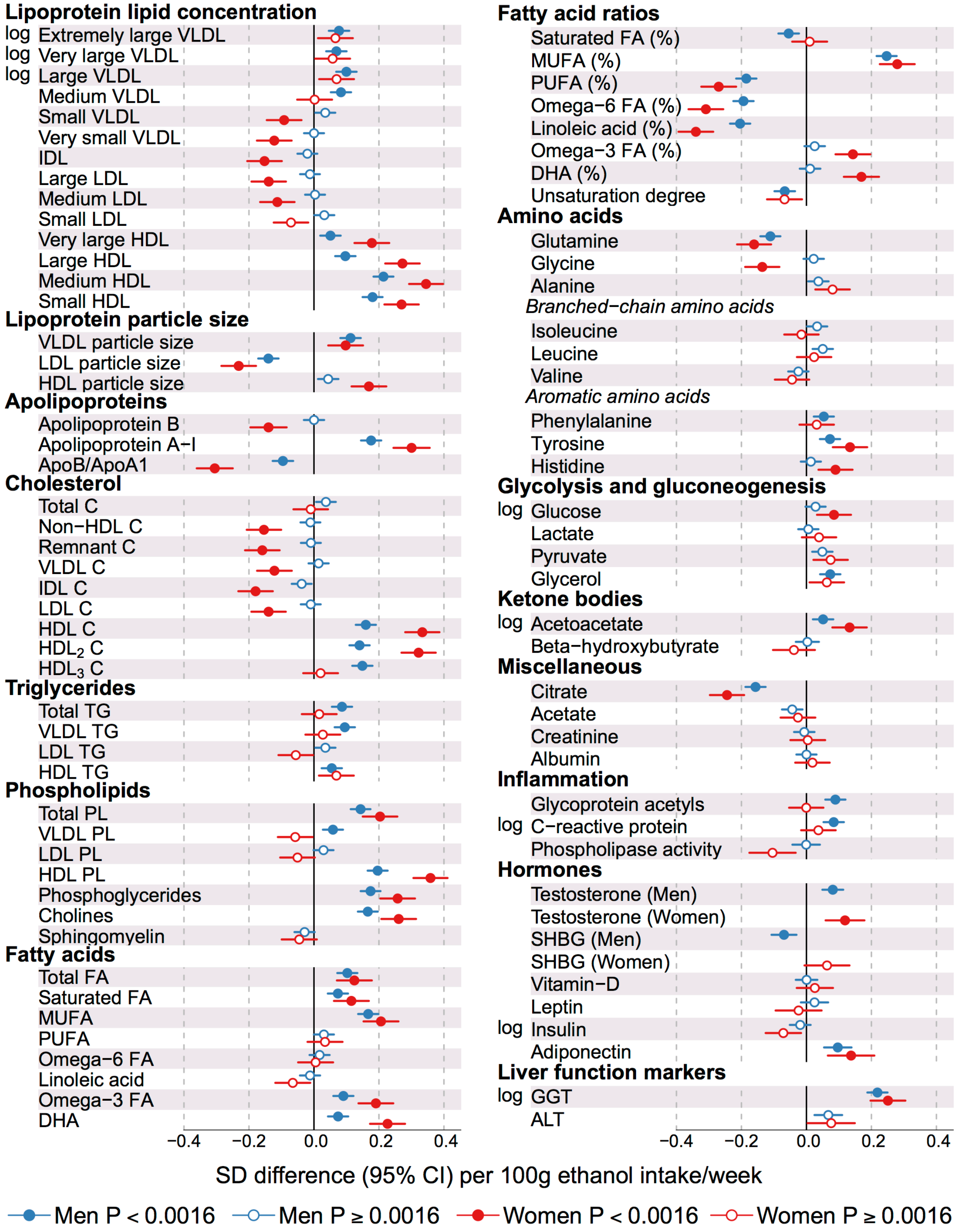

Sex-specific associations of alcohol consumption with metabolic for 4707 men (blue) and 5071 women (red). The associations were adjusted for age and meta-analysed for the three cohorts. Open and closed circles indicate P≥0.002 and P<0.002, respectively. Although the association magnitudes per 100g weekly ethanol intake are generally stronger for women, the Pearson's correlations are similar, because 1-SD ethanol intake is 91 g/week for men and 52 g/week for women. For instance, the correlation of alcohol with Medium HDL is r=0.19 for men and r=0.18 for women. Similarly, the correlation of alcohol with omega-6 fatty acid% is r=-0.18 for men and r=-0.16 for women.

Figure S5: Cross-sectional associations with additional adjustment for potential confounders.

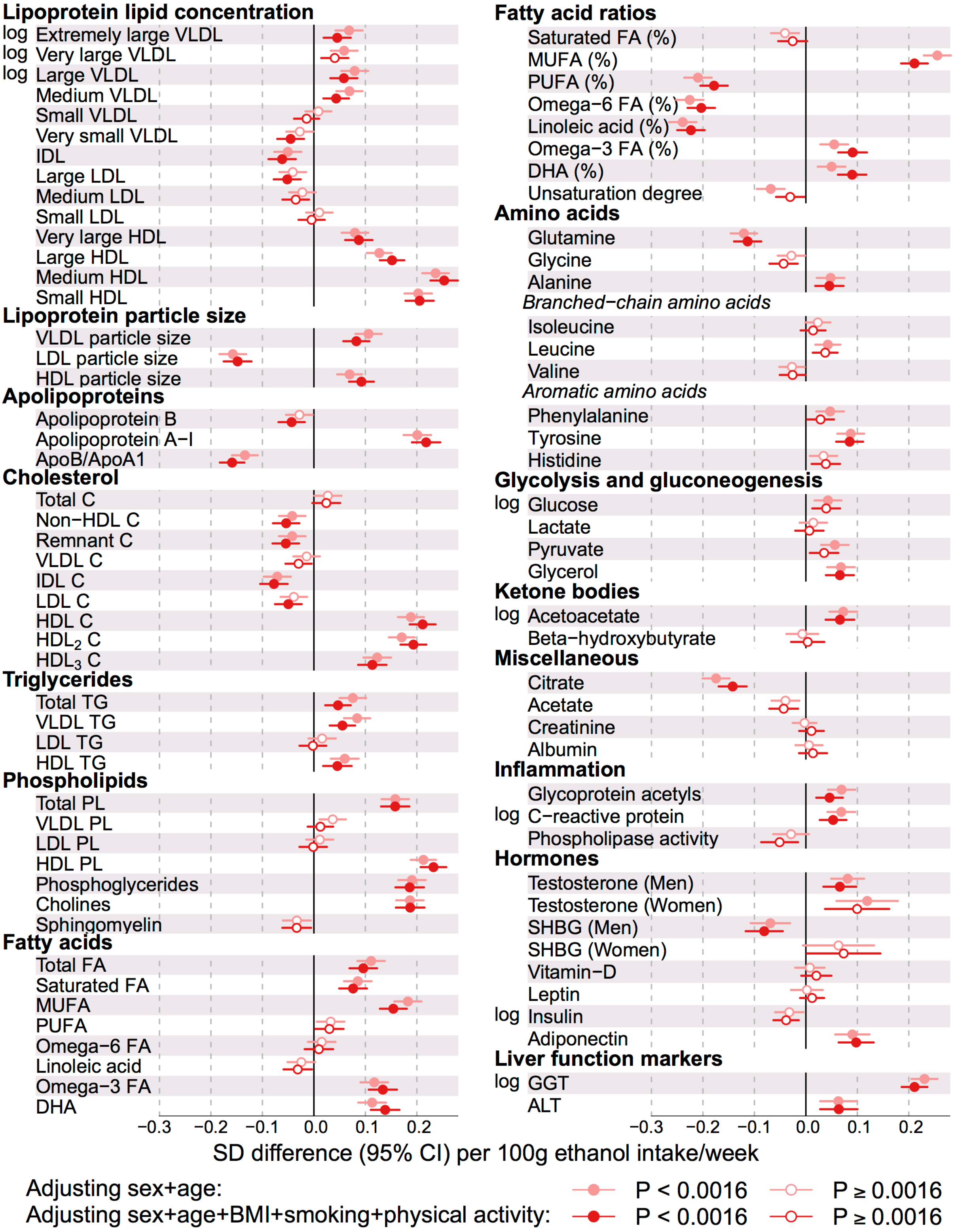

Cross-sectional associations between alcohol consumption and circulating metabolic measures without (upper) and with (lower) adjustment for BMI, smoking, and physical activity. Results are from meta-analyses of the three population-based cohorts (n=9778). All analyses were adjusted for age and sex.
